# Supplementary figures and images for: Application of the Sepsis-3 criteria to describe sepsis epidemiology in the Amsterdam UMCdb intensive care dataset
Source: PLoS One. 2024 Jun 21;19(6):e0304133. doi: 10.1371/journal.pone.0304133 (PMC11192388; doi:10.1371/journal.pone.0304133)

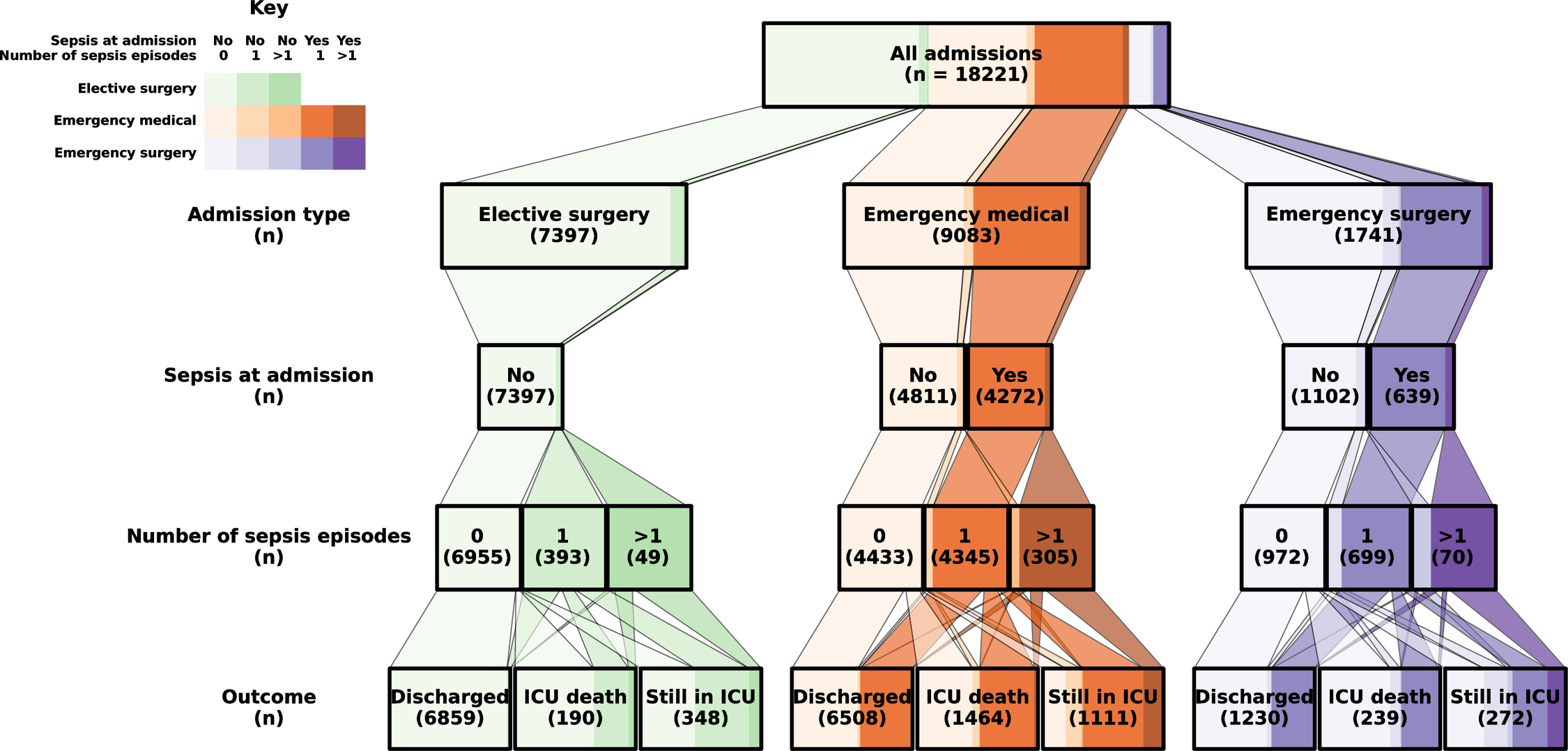

Supplement: S1 Fig — (TIF) [file pone.0304133.s001.tif]
